# Phenolic Compound Profiles in Berry Skins from Nine Red Wine Grape Cultivars in Northwest China

**DOI:** 10.3390/molecules14124922

**Published:** 2009-12-01

**Authors:** Zan-Min Jin, Jian-Jun He, He-Qiong Bi, Xiang-Yun Cui, Chang-Qing Duan

**Affiliations:** Centre for Viticulture and Enology, College of Food Science & Nutritional Engineering, China Agricultural University, Beijing 100083, China; E-Mails: zanminjin@yahoo.com.cn (Z-M.J.); hejj.email@gmail.com (J-J.H.); biheqiong0915@gmail.com (H-Q.B); cuixiangyuncxy@126.com (X-Y.C.)

**Keywords:** anthocaynins, non-anthocyanins, phenolic compounds, phenolic profile, wine grape

## Abstract

Phenolic compound profiles were investigated by HPLC-MS in two consecutive years to assess genotypic variation in berry skins of nine red *Vitis vinifera* cultivars. The results showed that the types and levels of phenolic compounds greatly varied with cultivar. Common wine grape cultivars such as Syrah, Cabernet Sauvignon, Cabernet Gernischt and Merlot contained more types of anthocyanins, flavonols, flavan-3-ols, stilbenes and phenolic acids than Gamay, Yan73, Pinot Noir, Zinfandel and Mycкат Poзoвый. Yan 73 and Pinot Noir had abundant anthocyanins, but only a few non-anthocyanin phenolic compounds. Gamay, Zinfandel and Mycкат Poзoвый contained only a few anthocyanins and flavonols. For a grape cultivar, the ratio of one anthocyanin content to total anthocyanin content did not change greatly from one year to the next, unlike for non-anthocyanins. Cluster analysis showed that except for Syrah and Yan 73, the phenolic profiles in the tested grape cultivars had no significant year-to-year variations.

## Introduction 

Wine is a widely consumed beverage in the world, with thousands of years of tradition. The phenolic compounds in grape berries are responsible for some of the major organoleptic properties of wine, such as color, astringency, bitterness, and aroma [1,2]. During the red winemaking process, phenolic compounds from the skins of red grapes transfer to the must during the fermentation and any maceration steps [3]. Based on their carbon skeleton, phenolic compounds are divided into two groups: flavonoid (anthocyanins, flavan-3-ols, flavonols) and non-flavonoid compounds (hydroxybenzoic and hydroxycinnamic acids, stilbenes). Different types of phenolic compounds endow grape varieties and wines with specific quality characteristics. 

As a material for winemaking, the phenolic compounds of wine grape are one of the most important aspects determining wine quality. In recent years, the phenolic profiles of wines with different geographical origin or with different varieties have been investigated, and these studies have shown that the phenolic profiles greatly depend on the origins or the varieties of the wines and thus could be used for the identification of wine geographical origin and variety [4,5,6,7,8]. Some research into the phenolic compounds of wine grapes have been reported, however, these studies have focused on the anthocyanin profiles in wine grapes from different varieties, as well as on comparison of total contents and antioxidant activities of phenolic compounds [9,10,11,12,13,14]. Little attention has been paid to non-anthocyanin phenolic compound profiles of different wine grape varieties. Actually, the phenolic compounds of wine grape berries vary strongly with variety/cultivar and environmental factors such as climate, soil, and cultural practices [2,15,16]. The influence of vine vigor on Pinot Noir anthocyanins and proanthocyanidins has been detailed by Cortell *et al*. [17,18]. Price *et al*. [19] and Spayd *et al*. [20] suggested that there is a positive correlation between sunlight exposure and an increase in flavonols. Downey *et al*. [21] also suggested that flavonoid biosynthesis in plants is affected by many factors, including light, temperature, altitude, soil type, water, microbial interactions and nutritional status. A detailed study of the phenolic compounds of wine grape is essential for evaluating the potential of the different grape varieties, optimizing enological processes to obtain products with the improved characteristics. 

The main aim of this study was to investigate the phenolic compounds characteristics of nine red grape cultivars (*Vitis vinifera* L.) from the foot of Qi-lian Mountain on the ancient Silk Road in northwest China, which would help to evaluate the winemaking potential of the different grape cultivars and provide some information for improving grape berry quality through appropriate cultivation models to regulate the effects of sunshine or water on phenolic compounds.

## Results and Discussion

### Anthocyanin compounds

**Figure 1:**
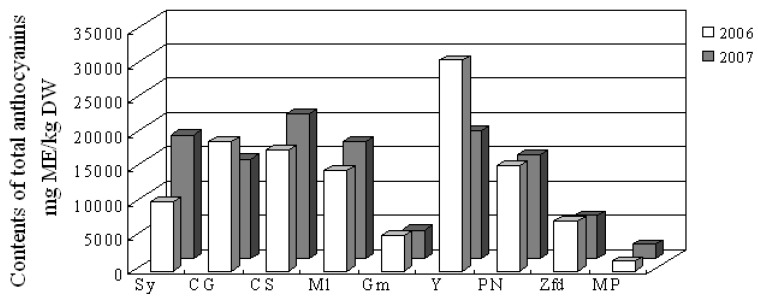
Total anthocyanin compound levels in wine grape skins.

Anthocyanins are mainly located in the grape skins and are largely responsible for the color of red wines. Figure 1 shows the content of total anthocyanin compounds in the skins from nine grape cultivars, which ranged from about 1,500 to 30,000 mg ME/kg DW. Anthocyanin levels in the skins of Syrah, Cabernet Gernischt, Cabernet Sauvignon, Merlot, Yan 73, and Pinot Noir were all significantly higher than those in the skins of Gamay, Zinfandel and Mycкат Poзoвый, while the skin of Mycкат Poзoвый had the lowest total anthocyanin content of all the cultivars tested. Moreover, such a cultivar difference in total anthocyanin content did not change with the year. It has been reported that there were no significant differences in the anthocyanin content of Cabernet Sauvignon and Merlot, which is consistent with our results [11,13]. As regards individual grape cultivars, except for Syrah and Yan 73, almost no significant difference in total anthocyanin content was observed between the years 2006 and 2007.

A total of eighteen anthocyanins were indentified from the skins of these *Vitis vinifera* grapes (Table 1). These anthocyanins were the monoglucosides and the monoglucoside derivatives of five anthocyanidins: delphinidin, cyanidin, petunidin, peonidin and malvidin [22]. Derivatives included 6-*O*-acetyl, 6-*O*-coumaryl, 6-*O*-caffeoyl and 6-*O*-feruloylglucoside. All the monoglucosides, acetyl monoglucosides and coumaryl monoglucosides of these five anthocyanidins could be observed in Cabernet Gernischt, Cabernet Sauvignon and Merlot, while hardly any acylated anthocyanins were found in Mycкат Poзoвый and Gamay. The main difference between Mycкат Poзoвый and Gamay was that the content of malvidin derivatives in Gamay was obviously higher than that in Mycкат Poзoвый. Among the anthocyanin compounds, pelargonidin-glucoside was found only in Yan 73 in 2006, with a content of about 50 mg ME/kg DW. Interestingly, this novel compound was also detected in the skins of Yan 73 grown in Yan-tai region of China (data not shown). As far as we know, Yan 73 belongs to *Vitis vinifera*, and no pelargonidin or its derivatives has been reported in the grapes of *Vitis vinifera* [23,24]. To verify this finding, further research on Yan 73 shall be performed. From the data in Table 1, it can be observed that the content of cyanidin-glucoside in the skins of Yan 73 and Pinot Noir were between 2,600~3,200 mg ME/kg DW and 1,200~1,400 mg ME/kg DW respectively, which were much higher than was found in the other grape cultivars tested (ranging from 30 to 400 mg ME/kg DW). The five monoglucoside anthocyanins made up over 60% of the total anthocyanins in all the samples, and the proportion of acetyl-anthocyanins was close to that of coumaryl-anthocyanins in the skins of Syrah, Cabernet Gernischt, Cabernet Sauvignon and Yan 73; however, the proportion of acetyl-anthocyanins was twice as much as that of coumaryl-anthocyanins in Merlot and Pinot Noir, which was opposite to the anthocyanin composition in the skins of Zinfandel. Cabernet Gernischt and Cabernet Sauvignon had no significant difference in anthocyanin composition.

### Flavonol compounds

**Figure 2:**
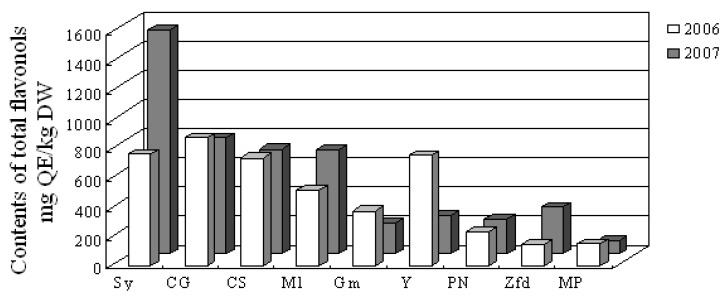
Total flavonol content in wine grape skins.

Flavonols are yellow pigments that mainly exist as the 3-*O*-glycoside of four main aglycones: myricein, quercetin, kaempherol and isorhamnetin [22]. They generally act as UV protectors [25,26], and co-pigments of anthocyanins in flowers and fruits [27,28]. As shown in Figure 2, the content of total flavonols in these wine grape skins ranged about from 90 to 1,500 mg QE/kg DW. Mycкат Poзoвый had the lowest total flavonol content. 

There was a great difference in the total flavonol content in the skins of Syrah and Yan 73 between 2006 and 2007. For Syrah, the content of flavonol in 2007 was about twice as much as that in 2006, and for Yan 73, the content of flavonol in 2006 was near three times as much as that in 2007. It also can be observed that the content of total flavonols in Syrah, Cabernet Gernischt, Cabernet Sauvignon and Merlot were noticeably higher than those in Gamay, Pinot Noir, Zinfandel and Mycкат Poзoвый.

Nineteen flavonol compounds in all were detected in these samples, and the composition of flavonols varied widely with grape cultivar (Table 2). Only quercetin-glucoside, quercetin-glucuronide and dihydroquercetin-glucoside were common in the skins of all grape cultivars. The greatest range of flavonol compounds was found in the skin of Syrah and Cabernet Sauvignon, followed by Merlot, Cabernet Gernischt, Yan 73, and Gamay. High levels of total flavonols were present in the skins of Syrah, Cabernet Gernischt and Cabernet Sauvignon, being mainly isorhamnetin-glucoside, myricetin-glucoside, syringetin-glucoside, and Merlot was rich in isorhamnetin-glucoside and myricetin-glucoside. In 2006 Yan 73 had elevated levels of quercetin-glucoside and myricetin-glucoside in the skin. Eight flavonol aglycones, quercetin, kaempferol, isorhamnetin, myricetin, laricitrin, syringetin, dihydroquercetin, dihydrokaempferol, were detected in Syrah, Cabernet Gernischt, Cabernet Sauvignon, Merlot, and Garmay. However, there were no dihydrokaempferol derivatives in Yan 73, no kaempferol, isorhamnetin and dihydrokaempferol derivatives in Pinot Noir, no laricitrin, isorhamnetin and dihydrokaempferol derivatives in Zinfandel, while only three aglycones, including quercetin, kaempferol and dihydroquercetin, were found in Mycкат Poзoвый. Among these grapes, dihydroquercetin-caffeoylglucoside only existed in Gamay, laricitrin-hexoside only in Merlot, and dihydroquercetin-hexoside only in Syrah. The content and composition of flavonols in Cabernet Gernischt were very close to those of Cabernet Sauvignon. As for each grape cultivar, except one or two compounds, other flavonol compounds remained almost stable in the years 2006 and 2007.

### Flavan-3-ols and non-flavonoid phenolic compounds

**Figure 3:**
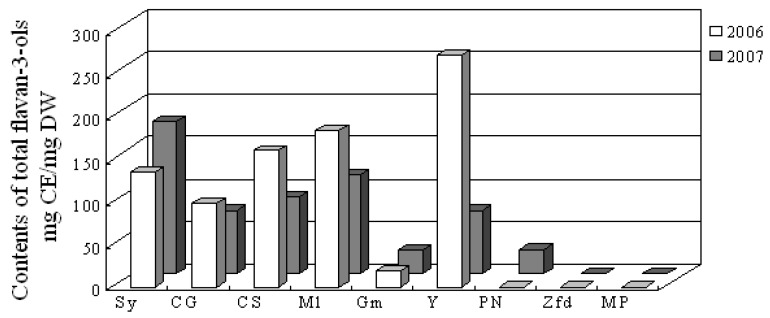
Contents of total flavan-3-ol compounds in wine grape skins.

The monomeric, oligomeric, or polymeric forms of flavan-3-ols which are found in both the seed and skin of the berry are responsible for the important wine astringency [22]. The content of total flavan-3-ol, as shown in Figure 3, varied greatly with the year and grape cultivar, indicating that flavan-3-ols are prone to the impact of environmental factors. The total flavanol content in Syrah, Cabernet Gernischt, Cabernet Sauvignon, Merlot and Yan 73 were significantly higher than those in Gamay and Pinot Noir, while no flavan-3-ol and non-flavonoid phenolic compounds were identified in Zinfandel and Mycкат Poзoвый (Table 3). Also, only one flavan-3-ol, catechin, and one non-flavonoid, *trans*-piceid, were found in the skin of Gamay. In the skins of Pinot Noir sampled in 2007, only a little amount of catechin and proanthocyanidin dimer was detectable. 

### Cluster analysis 

**Figure 4:**
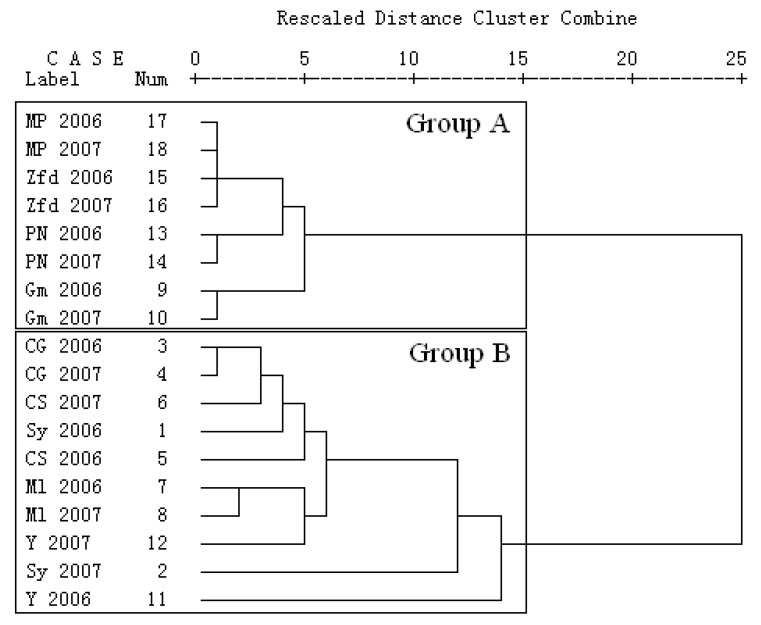
Cluster analysis of the nine grape cultivars from Qi-lian region of China.

To better understand the characteristics of phenolic compounds from these nine grape cultivars, cluster analysis was carried out on the data for the identified compounds. The squared Eulidean distance was taken as a measure of proximity between two grape samples, and Ward’s method was used as the linkage rule. As shown in Figure 4, the nine grape cultivars were clearly divided into two groups in the dendrogram: Group A, including Mycкат Poзoвый, Zinfandel, Pinot Noir and Gamay, and Group B, including Cabernet Gernischt, Cabernet Sauvignon, Syrah, Merlot and Yan 73. In Group A, Mycкат Poзoвый, Zinfandel were clustered within a short distance, which corresponds to their similar phenolic profiles. As seen in the above data, both Mycкат Poзoвый and Zinfandel had only a few flavonols, flavan-3-ols and non-flavonoid compounds. Compared with these two cultivars, Pinot Noir and Gamay had a relatively large cluster distance, which may be related to the fact that the composition and content of anthocyanins in Pinot Noir were significantly higher than those in Gamay although they had similar the composition and content of non-anthocyanins phenolic compounds. With regards to Group A and Group B, the grape cultivars in Group A all contained less phenolic compounds than those in Group B. Besides, the cluster analysis also indicated that for any one cultivar in Group A, the phenolic profile in 2006 was similar to that in 2007. 

However in Group B only Cabernet Gernischt and Merlot were clustered within a short distance, showing minor difference in phenolic profiles between 2006 and 2007. In contrast there was a large cluster distance in between phenolic compounds in Yan 73 berries for 2006 and 2007, and a similar result was also found for the phenolic compounds in Syrah berries for 2006 and 2007, suggesting that the phenolic accumulation in both Yan 73 and Syrah was greatly affected by climate. According to data in Table 1, Table 2 and Table 3, the significant variation for Yan 73 between 2006 and 2007 was mainly in the phenolic content and not in the composition, while the great variation in Syrah between 2006 and 2007 was in both the composition and in the content of phenolic compounds.

## Experimental

### Chemicals and standards 

The malvidin-3-o-glucoside standard was purchased from Extrasynthese SA (Genay, France), the catechin, quercetin, gallic aid, cafteic acid, and resveratrol standards were all purchased from Sigma Company (Iowa, USA). HPLC grade methanol, formic acid, acetic acid and acetonitrile were obtained from Fisher Company (Fairlawn, NJ, USA). Ethyl acetate (analytical grade) was obtained from Beijing Chemical Reagent Plant (Beijing, China). Deionized water (<18 MΩ resistance) was obtained from a Milli-Q Element water purification system (Millipore, Bedford, MA, USA).

### Wine grape samples preparation

The nine red wine grape cultivars, Syrah, Cabernet Sauvignon, Cabernet Gernischt, Merlot, Gamay, Yan 73, Pinot Noir, Zinfandel, Mycкат Poзoвый, were collected from the foot of Qi-lian Mountain on the ancient Silk Road in northwest China in 2006 and the collection was repeated in 2007. The average altitude of the vinyard is 1,214 meters. Cabernet Gernischt (*V. vinifera* L.) is a Chinese characteristic cultivar that might have been imported from Europe in 1894 and naturally selected in China. Yan 73 is a dyer cultivar and was obtained from the hybridization of Alicante Bouschet (*V. vinifera* L.) as female parent and Muscat Hamburg (*V. vinifera* L.) as male parent in 1966. Mycкат Poзoвый is a variant of Muscat Blanc à Petits Grains that is a member of the Muscat family of *Vitis vinifera*. It is known under a variety of local names such as Muscat rouge de Frontignan (France), Moscato rosso di Madèra (Italian), Mycкат кpacный and Mycкат Фpoнтиньянcкий (Crimea). It is more prevalent in Crimea and was imported from Romania to China in 1955. All these cultivars were planted in the same area, with similar climatic conditions and soil characteristics. These cultivars were subjected to the same management practice, such as irrigation, fertilization, soil management, disease control and pruning. The grape berries were harvested at technological ripeness, determined on the basis of former years’ ripening dates and as judged from seed color change to dark brown without senescence of berry tissue. To obtain a sample representing a vineyard population, we sampled according to the method described by Boulton *et al*. [29]. Three 100-berry samples were selected from at least seven 10-cluster selections at similar position of 30 whole vine selections. The fresh samples were kept in refrigerated bags, and taken to the laboratory within a few hours. Then the grape skins were peeled from berries and frozen in the liquid nitrogen followed by lyophilization and grinding. Grape skin powders were stored at −40 °C until use.

### Extraction and analysis of anthocaynins

Grape skin powder (1.00 g) was immersed in methanol (20 mL) containing 1% formic acid. The extraction was performed with the aid of ultrasonic vibration for 10 min, and then shaking in the dark at 25 °C for 30 min at a rate of 150 rpm. The homogenate was centrifuged at 8,000 × *g* for 20 min and the supernatant was collected. The residues were re-extracted four times. All the supernatants were mixed, concentrated to dryness using a rotary evaporator and then redissolved in 10 mL Chinese white wine. The resulting suspensions were filtered through 0.45 µm filters (cellulose acetate and nitrocellulose, CAN) prior to HPLC-MS analysis.

Qualitive and quantitive analyses of anthocyanin extracts were performed as reported in our previous work [30] using an Agilent 1100 series LC-MSD trap VL instrument equipped with a Diode Array Detector (DAD) and reverse phase column (Kromasil C_18_, 250 × 4.6 mm, 5 μm). Compounds were gradient eluted using two solutions as follows: (A) aqueous 2% formic acid, and (B) acetonitrile containing 2% formic acid. The gradient was from 6% to 10% B over 4 min, from 10% to 25% B over 8 min, isocratic 25% B over 1 min, from 25% to 40% over 7 min, from 40% to 60% over 15 min, from 60% to 100% over 5 min, and from 100% to 6% over 5 min, at a flow rate of 1.0 mL min^-1^. Injection volumes were 30 μL, and the detection wavelength was 525 nm. MS conditions were as follows: Electrospray ionisation (ESI) interface, positive ion model, 35 psi nebulizer pressure, 10 mL min^-1^ dry gas flow rate, 350 ºC dry gas temperature, and scans at *m/z* 100–1,000. All analyses were replicated twice.

### Extraction and analysis of non-anthocaynins 

Grape skin powder (5.00 g) was macerated with distilled water (10 mL) and then after the addition of ethyl acetate (50 mL) the mixture was shaken in darkness for 30 min. The residue was extracted repeatedly five times; while all the supernatants were mixed, concentrated to dryness using a rotary evaporator, redissolved in methanol (5 mL), and finally filtered through 0.22 µm Nylon membrane filters for HPLC-MS analysis later [31].

An Agilent 1200 series HPLC-MSD trap VL instrument, equipped with a Variable Wavelength Detector (VWD) and a reverse phase column (Zorbax SB-C_18_ column 3 × 50 mm, 1.8 µm), was used for separation of non-anthocyanin phenolic compounds. Compounds were gradient eluted using two solutions as follows: (A) aqueous 1% acetic acid, and (B) acetonitrile containing 1% acetic acid. The gradient was 5% to 8% B for 10 min, 8% to 10% B for 8 min, 10% to 15% for 22 min, 15% to 20% for 10 min, 20% to 30% for 3 min, 30% to 50% for 5 min, 50% to 100% for 4 min, and isocratic 100% B for 4 min,, at a flow rate of 1.0 mL min^-1^. Injection volumes were 2 μL and the detection wavelength was 280 nm. MS conditions were as follows: Electrospray ionisation (ESI) interface, negative ion model, 35 psi nebulizer pressure, 10 mL min^-1^ dry gas flow rate, 325 ºC dry gas temperature, and scans at *m/z* 100–1,000. All analyses were replicated twice.

### Statistical analysis 

Anthocyanins, flavanols, flavonols, hydroxybenzoic acids, hydroxycinnamic acids and stilbenes were quantified and expressed as mg of malvidin-3-O-glucoside equivalents (ME), catechin equivalents (CE), quercetin equivalents (QE), gallic acid equivalents (GAE), cafteic acid equivalents (CAE) and resveratrol equivalents (RE) per kg of dry weight of grape skin, respectively. Analysis of variance was performed with One-Way ANOVA procedure of SPSS for windows (v. 11.5.), and data were shown as means ± standard deviation (S.D.) of three independent experiments with two replicates. The average values of the obtained data set were subjected to cluster analysis.

## Conclusions

To summarize, there was great variability in the composition and content of phenolic compounds in the grape berry skin between the grape cultivars. In general, Syrah, Cabernet Sauvignon, Cabernet Gernischt and Merlot were rich in various phenolic compounds, including anthocyanins, flavonols, flavan-3-ols, stilbenes and phenolic acids. Yan 73 and Pinot Noir also contained abundant anthocyanins, but the types and contents of non-anthocyanin phenolic compounds of Yan 73 varied greatly from one year to the next. The skin of Pinot Noir exhibited fewer non-anthocyanin phenolic compounds. Only a few anthocyanins and flavonols were detected in the skins of Zinfandel and Mycкат Poзoвый. The phenolic compounds in Syrah and Yan 73 seemed to be more easily affected by the year, compared with the other grape cultivars. Moreover, the proportion of individual anthocyanin content to total anthocyanin content varied with grape cultivar, but generally did not differ greatly for a given cultivar from one year to the next.

## Figures and Tables

**Table 1:** The contents of anthocyanins compounds in the berry skins from nine grape cultivars (mg ME/kg DW).

	Sy		CG		CS		Ml		Gm		Y		PN		Zfd		MP	
	2006	2007	2006	2007	2006	2007	2006	2007	2006	2007	2006	2007	2006	2007	2006	2007	2006	2007
Dp-glu	1097±11	1660±13	2203±17	1294±12	2057±14	1988±15	1674±13	2059±13	200±3	138±2	6972±19	4380±15	2373±12	2272±11	745±6	476±4	48±2	63±2
Cy-glu	111±2	240±3	309±3	135±2	43±1	38±1	308±3	363±2	57±1	46±1	3118±12	2650±9	1325±	1233±21	243±8	240±2	41±1	49±1
Pt-glu	987±5	1616±12	1616±11	1055±	1558±12	1867±5	1217±15	1470±20	259±4	211±4	4910±25	3613±32	1429±11	1596±23	735±8	558±3	52±2	77±2
Pn-glu	679±3	1273±6	894±4	530±4	847±6	1165±6	1080±7	1295±10	534±3	467±6	3342±33	1235±16	1528±8	1491±7	706±9	855±7	307±4	527±2
Mv-glu	3579±14	7395±21	6929±23	5834±17	6438±19	8754±23	5873±16	6835±15	3847±11	3058±12	7459±18	3837±14	6019±13	5804±15	3579±12	3223±11	1022±7	1377±5
Pl-glu	nd	nd	nd	nd	nd	nd	nd	nd	nd	nd	54±	nd	nd	nd	nd	nd	nd	nd
Dp-ac	164±2	306±2	492±2	285±1	502±2	760±3	376±2	450±2	nd	nd	624±3	401±3	337±2	371±1	45±1	nd	nd	nd
Cy-ac	nd	nd	110±1	74±1	134±1	189±2	121±1	133±2	nd	nd	323±2	270±2	90±2	78±2	nd	nd	nd	nd
Pt-ac	231±3	336±2	397±3	257±2	408±3	536±4	297±3	350±4	nd	nd	570±4	435±3	274±2	251±2	44±1	36±1	nd	nd
Pn-ac	142±2	254±2	191±1	120±2	179±2	240±3	268±2	286±3	nd	nd	nd	nd	288±2	268±2	103±2	69±2	nd	nd
Mv-ac	1303±6	1985±11	1944±11	1681±12	1828±9	2497±14	1737±8	1813±8	131±2	94±1	941±5	428±3	901±4	887±5	172±2	152±1	nd	53±1
Dp-co	nd	nd	425±2	268±2	376±4	500±4	157±2	201±1	nd	nd	787±7	426±6	nd	nd	nd	nd	nd	nd
Cy-co	nd	nd	152±1	177±2	77±1	112±1	128±2	254±2	nd	nd	178±2	127±2	nd	nd	nd	nd	nd	nd
Pt-co	nd	nd	393±3	277±2	315±2	418±3	135±1	169±2	nd	nd	372±2	269±2	142±1	129±1	102±2	61±1	nd	nd
Pn-co	288±2	502±2	308±2	239±3	328±2	426±3	188±1	218±2	37±1	29±2	236±3	112±2	88±1	93±2	114±2	124±2	nd	nd
Mv-co	1629±9	2520±12	2503±13	2280±13	2380±11	1689±8	1041±10	1202±9	189±3	136±2	835±8	441±4	597±3	608±3	751±4	502±2	31±1	nd
Pn-ca	nd	nd	40±1	43±1	39±1	nd	nd	nd	nd	nd	nd	nd	nd	nd	nd	nd	nd	nd
Mv-ca	nd	nd	nd	nd	86±1	nd	nd	nd	nd	nd	nd	nd	nd	nd	nd	nd	nd	nd
Mv-fe	nd	nd	nd	nd	59±1	nd	nd	nd	nd	nd	nd	nd	nd	nd	nd	nd	nd	nd

Abbreviation: Dp, delphinidin; Cy, cyanidin; Pt, petunidin; Pn, peonidin; Mv, malvidin; Pl, pelargonidin; glu, glucoside; ac, acetylglucoside; co, coumarylglucoside; ca, caffeylglucoside; fe, feruloylglucoside.

**Table 2:** The contents of flavonols compounds in the berry skins from nine grape cultivars (mg QE/kg DW).

	Sy		CG		CS		Ml		Gm		Y		PN		Zfd		MP	
	2006	2007	2006	2007	2006	2007	2006	2007	2006	2007	2006	2007	2006	2007	2006	2007	2006	2007
Q-glu	nd	358.6±3.7	31.3±2.1	32.6±1.3	30.5±1.7	76.7±2.0	40.2±2.9	67.4±2.6	20.6±2.3	10.3±1.4	112.8±3.2	43.7±2.1	22.1±2.2	28.5±2.8	16.3±2.3	11.5±2.9	44.3±3.1	21.1±2.7
K-glu	70.0±2.6	118.3±4.3	63.7±2.5	76.8±2.9	50.7±2.4	71.4±2.4	39.7±1.3	65.9±2.3	nd	nd	27.1±2.2	nd	nd	nd	nd	27.7±2.3	23.3±2.2	11.0±2.1
IR-glu	245.7±3.4	265.6±3.6	283.3±3.4	285.6±3.7	214.1±2.3	91.7±1.2	123.5±2.7	134.8±4.0	52.5±2.5	46.7±2.3	76.7±3.2	nd	nd	nd	nd	nd	nd	nd
M-glu	135.2±4.1	181.8±3.8	168.9±2.5	129.8±2.3	139.0±1.9	95.4±1.6	149.4±1.7	150.0±2.8	13.8±2.2	nd	237.9±3.3	88.6±3.2	15.6±2.3	88.1±2.2	49.8±1.3	38.1±2.3	nd	nd
L-glu	35.3±2.2	35.0±2.3	39.1±2.2	29.4±2.1	32.3±2.3	22.9±3.1	23.2±2.7	28.3±2.6	9.7±2.4	9.5±2.6	35.0±2.7	13.1±2.2	nd	16.9±2.5	nd	nd	nd	nd
S-glu	120.3±2.7	107.9±2.4	144.2±2.8	128.3±1.8	143.7±2.0	136.3±2.1	47.5±2.5	82.2±2.4	42.9±2.7	46.1±3.2	63.7±2.9	37.0±3.7	nd	35.3±3.2	nd	166.3±3.2	nd	nd
Q-gal	14.6±1.2	56.2±1.4	8.2±2.3	8.3±2.1	nd	16.7±2.4	nd	13.5±1.4	nd	nd	15.5±2.0	nd	nd	nd	nd	nd	7.7±1.6	nd
K-gal	nd	34.3±2.6	17.7±2.6	nd	nd	10.5±1.7	nd	nd	25.2±2.7	12.4±2.5	11.3±2.3	nd	nd	nd	nd	nd	nd	nd
IR-gal	nd	60.8±2.3	nd	nd	nd	nd	nd	nd	nd	nd	nd	nd	nd	nd	nd	nd	nd	nd
Q-gcn	43.2±2.3	155.7±2.9	26.4±2.3	32.4±2.6	22.1±2.8	61.3±1.7	27.0±2.4	58.9±2.5	28.3±2.3	30.7±2.2	55.2±2.7	42.9±2.6	62.3±2.7	31.7±2.4	55.4±2.7	53.5±2.6	70.5±3.2	61.7±2.7
DQ-rha1	25.3±2.2	51.3±2.6	20.0±2.4	19.5±2.8	33.0±1.9	50.1±3.2	8.7±2.3	12.9±2.1	99.9±2.0	23.9±2.0	12.8±2.2	nd	93.1±3.3	16.6±2.3	27.5±2.3	23.2±2.4	7.1±2.6	nd
DQ-rha2	24.5±2.3	41.1±2.7	nd	nd	nd	nd	nd	nd	nd	nd	nd	nd	20.7±2.7	nd	nd	nd	nd	nd
DK-rha	18.7±2.7	14.0±2.4	14.8±1.9	12.2±1.6	12.5±0.9	16.7±1.2	nd	10.7±1.7	33.5±1.9	24.7±2.1	nd	nd	nd	nd	nd	nd	nd	nd
L-ac	nd	nd	nd	nd	nd	30.4±2.3	20.3±2.3	28.0±2.7	nd	nd	22.6±2.6	17.0±3.0	nd	nd	nd	nd	nd	nd
DQ-ca	nd	nd	nd	nd	nd	nd	nd	nd	20.1±1.4	10.0±0.7	nd	nd	nd	nd	nd	nd	nd	nd
M	nd	nd	36.7±1.6	17.3±1.2	24.6±1,6	9.8±1.0	28.9±1.1	21.0±1.8	nd	nd	49.5±1.9	19.9±1.5	nd	nd	nd	nd	nd	nd
L-h	nd	nd	nd	nd	8.1±	nd	11.1±2.3	11.4±1.8	nd	nd	nd	nd	nd	nd	nd	nd	nd	nd
DQ-h1	14.4±2.0	24.0±1.9	nd	nd	nd	nd	nd	nd	nd	nd	nd	nd	nd	nd	nd	nd	nd	nd
DQ-h2	18.4±2.0	24.9±1.6	26.4±1.8	23.6±2.1	28.8±2.1	26.3±2.3	nd	29.0±1.7	29.8±2.3	nd	37.3±2.4	nd	19.8±2.4	23.8±2.1	nd	nd	nd	nd

Abberviatives: Q, quercetin; K, kaempferol; IR, isorhamnetin; M, myricetin; L, laricitrin; S, syringetin; DQ, dihydroquercetin; DK, dihydrokaempferol; glu, glucoside; gal, galactoside; gcn, glucuronide; rha, rhamnoside; ac, acetylglucoside; ca, caffeoylglucoside; h, hexoside.

**Table 3:** The contents of flavan-3-ol and non-flavonoid phenolic compounds in the berry skins from nine grape cultivars (mg/kg DW).

	Sy		CG		CS		Ml		Gm		Y		PN		Zfd		MP	
	2006	2007	2006	2007	2006	2007	2006	2007	2006	2007	2006	2007	2006	2007	2006	2007	2006	2007
C	34.5±2.8	34.2±2.1	16.8±1.9	15.3±2.0	23.5±1.7	15.9±2.2	13.1±2.3	12.6±2.1	19.2±1.7	28.5±1.3	44.6±2.1	19.0±2.1	nd	8.5±0.9	nd	nd	nd	nd
EC	nd	nd	15.9±2.6	nd	nd	nd	37.4±3.2	25.1±2.9	nd	nd	79.5±2.4	nd	nd	nd	nd	nd	nd	nd
GC-C	nd	nd	5.4±1.5	8.2±2.2	9.8±1.5	9.3±1.9	16.5±1.2	17.9±1.4	nd	nd	27.2±1.4	16.4±1.5	nd	nd	nd	nd	nd	nd
P2a	22.1±1.8	40.7±3.0	15.6±2.4	14.3±2.1	31.8±2.3	20.5±2.3	36.3±2.3	18.4±2.1	nd	nd	27.6±1.9	nd	nd	20.1±2.3	nd	nd	nd	nd
P2b	11.4±2.0	18.6±2.6	7.4±1.7	11.3±2.3	11.2±2.0	nd	18.3±1.6	19.1±1.9	nd	nd	37.4±2.3	nd	nd	nd	nd	nd	nd	nd
P3	67.0±3.2	85.3±3.2	36.7±2.8	39.7±2.8	84.1±2.3	44.6±1.6	62.4±2.7	22.6±2.0	nd	nd	55.8±1.3	39.2±1.2	nd	nd	nd	nd	nd	nd
trans-P	nd	nd	18.4±1.7	15.9±2.0	6.9±1.8	5.2±1.3	25.6±2.4	28.1±2.3	17.8±1.2	6.2±2.3	26.8±2.8	9.7±1.9	nd	nd	nd	nd	nd	nd
cis-P	nd	nd	nd	nd	nd	nd	13.1±2.1	nd	nd	nd	nd	nd	nd	nd	nd	nd	nd	nd
HEVA	1.9±0.3	2.9±06	4.3±1.3	2.4±1.0	nd	3.6±1.2	2.5±0.9	tr	tr	tr	9.0±2.0	3.2±1.2	nd	nd	nd	nd	nd	nd
FA	nd	nd	nd	nd	nd	nd	nd	nd	nd	nd	17.2±1.5	14.1±1.8	nd	nd	nd	nd	nd	nd

Abbreviatives: C, Catechin; EC, Epicatechin; GC-C, (epi)Gallocatechin-(epi)Catechin; P2, Proanthocyanidin dimer; P3, Proanthocyanidin trimer; P, Piceid; HEPA, Hexose ester of protocatechuic acid; HEVA, Hexose ester of vanillic acid; FA, Ferulic acid.

## Abbreviations

Sy, Syrah; CG, Cabernet Gernischt; CS, Cabernet Sauvignon; Ml, Merlot; Gm, Gamay; Y, Yan 73; PN, Pinot Noir; Zfd, Zinfandel; MP, Mycкат Poзoвый.
